# The association between HPV gene expression, inflammatory agents and cellular genes involved in EMT in lung cancer tissue

**DOI:** 10.1186/s12885-020-07428-6

**Published:** 2020-09-24

**Authors:** Marzieh Rezaei, Shayan Mostafaei, Amir Aghaei, Nayyerehalsadat Hosseini, Hassan Darabi, Majid Nouri, Ashkan Etemadi, Andrew O’. Neill, Javid Sadri Nahand, Hamed Mirzaei, Seamas C. Donnelly, Mohammad Doroudian, Mohsen Moghoofei

**Affiliations:** 1 Department of Biology, Science and Research Branch, Islamic Azad University, Tehran, Iran; 2 Clinical Research Development Center, Imam Reza Hospital, Nurse Blvd, Kermanshah, Iran; 3 Inflammation Research Center, Tehran University of Medical Sciences, Tehran, Iran; 4 Medical Genetics Research Center, Department of Medical Genetics, Faculty of Medicine, Mashhad University of Medical Sciences, Mashhad, Iran; 5 AJA University of Medical Sciences, Golestan Hospital Research Center, Tehran, Iran; 6 Department of Biology, Faculty of Science, Shahrekord University, Shahrekord, Iran; 7 Department of Medicine, Trinity Centre, Tallaght University Hospital, Dublin 24, Ireland; 8 Department of Virology, Faculty of Medicine, Iran University of Medical Sciences, Tehran, Iran; 9 Biochemistry and Nutrition Research Center, Kashan University of Medical Sciences, Kashan, Iran; 10 Department of Cell and Molecular Biology, Faculty of Biological Sciences, Kharazmi University, Tehran, Iran; 11 Department of Microbiology, Faculty of Medicine, Kermanshah University of Medical Sciences, PO Box 6716777816, Razi Blvd, Kermanshah, Iran; 12 Medical Biology Research Center, Institute of Health and Technology, Kermanshah, University of Medical Sciences, Kermanshah, Iran

**Keywords:** Human papilloma virus, Lung Cancer, Tumour development, Inflammatory cytokines, Epithelial-mesenchymal transition (EMT)

## Abstract

**Background:**

Lung cancer is a leading cause of cancer morbidity and mortality worldwide. Several studies have suggested that Human papillomavirus (HPV) infection is an important risk factor in the development of lung cancer. In this study, we aim to address the role of HPV in the development of lung cancer mechanistically by examining the induction of inflammation and epithelial-mesenchymal transition (EMT) by this virus.

**Methods:**

In this case-control study, tissue samples were collected from 102 cases with lung cancer and 48 controls. We examined the presence of HPV DNA and also the viral genotype in positive samples. We also examined the expression of viral genes (E2, E6 and E7), anti-carcinogenic genes (p53, retinoblastoma (RB)), and inflammatory cytokines in HPV positive cases.

**Results:**

HPV DNA was detected in 52.9% (54/102) of the case samples and in 25% (12/48) of controls. A significant association was observed between a HPV positive status and lung cancer (*OR = 3.37, 95% C.I = 1.58–7.22, P = 0.001*). The most prevalent virus genotype in the patients was type 16 (38.8%). The expression of p53 and RB were decreased while and inflammatory cytokines were increased in HPV-positive lung cancer and HPV-positive control tissues compared to HPV-negative lung cancer and HPV-negative control tissues. Also, the expression level of E-cad and PTPN-13 genes were decreased in HPV- positive samples while the expression level of SLUG, TWIST and N-cad was increased in HPV-positive samples compared to negative samples.

**Conclusion:**

Our study suggests that HPV infection drives the induction of inflammation and EMT which may promote in the development of lung cancer.

## Background

Lung cancer is one of the leading causes of cancer morbidity and mortality worldwide [1]. There are several types of primary lung cancer which, are divided into two main groups; small cell lung cancer (SCLC) and non-small cell lung cancer (NSCLC). NSCLC are divided into three common types; squamous cell carcinoma, large cell carcinoma and adenocarcinoma [2]. The pathogenesis of lung cancer is a complex multifactor process with both genetic and environmental factors playing a major role [3]. Infectious agents are emerging as key drivers in the development of cancer [4–7]. Previously, numerous infectious agents have been shown to be involved in a myriad of lung diseases including cancer, Idiopathic Pulmonary Fibrosis (IPF) and Chronic Obstructive Pulmonary Disease (COPD) [8–10].

Human papilloma virus (HPV) is one of the most important human oncogenic viruses [11], which has previously been shown to be associated with numerous cancers including lung, breast and prostate [1, 6, 11–13]. The HPV genome is divided into three main sections; long control region (LCR), early region (E) encoding *E1, E2, E4–E7*, and late region (L) consisting of *L1* and *L2* [14]. E6 and E7 are the oncoproteins that act as stimulating factors for host cell proliferation [15]. E6 interacts with p53 and BCL2, while E7 interacts with retinoblastoma (RB); both of which lead to enhanced cell proliferation, resistance to apoptosis and chromosomal instability [16, 17]. These viral proteins enhance tumour development by promoting inflammation and epithelial-mesenchymal transition (EMT) [18, 19].

In response to harmful stimuli and invading pathogens, the innate immune system becomes activated through a variety of receptors, leading to the generation of an acute inflammatory response. This inflammation aids in the removal and clearance of the stimulus. However, should the stimulus fail to be removed the development of chronic inflammation occurs which is strongly associated with cancer [20].

Chronic inflammation as a result of viral infection is responsible for an estimated 25% of all human cancers [21, 22]. In response to viral infection the generation of a pro-inflammatory response involves activation of numerous transcription factors including NF-κB and the secretion of numerous pro-inflammatory cytokines and metabolites including transforming growth factors like beta (TGF-β), interleukin 1 (IL-1), IL-6, IL-11, Tumour necrosis factor α (TNF-α) and reactive oxygen-nitrogen species (RONS) - all of which play a pro-tumorigenic role in the context of chronic inflammation. This pro-inflammatory tissue microenvironment results in the suppression of anti-humoral immunity and also the promotion of tumour development and metastasis [7, 23, 24].

The second facet of high-risk HPV (hr-HPV) related tumour development is EMT, which plays an important role in solid cancer progression through multiple biochemical changes. EMT is well known to enhance cell migration, invasion and cancer development [25].

There are several genes involved in EMT, including SLUG, PTPN13, E-cad, N-cad and TWIST. SLUG protein is involved in important cellular events including EMT and also has anti-apoptotic activity [26]. PTPN13 interacts with Fas receptor which is indirectly involved in inhibition of programmed cell death [27]. E-cad and N-cad expression levels have also been connected with survival mechanisms and metastasis of lung cancer cells [28, 29].

In this study we investigated, for the first time, the role of hr-HPV in EMT and lung tumour development. We also assessed the prevalence of HPV in lung tumour samples; examining the expression level of viral and cellular genes and the associations between these expressed genes in EMT and lung tumour development.

## Methods

### Study design and samples

This case-control study was conducted between November 2017 and September 2018. One hundred and two lung cancer samples and forty-eight normal lung tissue samples. Control samples were age and sex matched, with the tissue samples collected from a peripheral region of the surgically removed lung cancers and non-cancer patients with fibrosis. All samples, cases and controls, were fresh tissue with a Tumor Proportion Score (TPS) > 50%. Control samples were age and sex matched. The TNM system was used to denote the stage of cancer as decided by a consultant oncologist and oncological surgeon. Gender, age, smoking status, tumour type and tumour stage were clinical parameters of patients that are shown in (supplementary materials). We had no medical records of HPV infection before cancer diagnosis.

### Extraction of nucleic acids

Total DNA extraction from tissue samples was performed by QIAamp® DNA Mini Kit (Qiagen, Hilden, Germany). Quality of extracted DNA was assessed by conducting PCR for *β-globin* as described before [30]. All samples were deemed suitable for molecular analysis due to β-globin gene amplification.

Total RNA extraction was conducted by RNeasy Mini Kit (Qiagen, Hilden, Germany).

### HPV detection and genotyping

HPV genome detection was conducted using PCR for *L1* and *E7* genes [31]. HPV genotyping was performed by INNO-LiPA HPV Genotyping v2 test (Innogenetics, Ghent, Belgium).

### Determination of HPV genome physical status

To determine the physical status of the HPV genome, the *E2*/*E6* ratio was used. An *E2*/*E6* ratio > 0 and < 1 indicates that the virus is in a mixed physical state, with both episomal and integrated forms of the virus [32].

### Quantitative real-time PCR

#### mRNA level detection of viral genes

Total RNA was extracted and purified from the tissue by using RNEasy Mini kit (QIAGEN, Hilden, Germany). For cDNA synthesis, 1 μg of total RNA was reverse transcribed using the QuantiNova Reverse Transcription Kit (QIAGEN, Germany). CDNA synthesis was performed in a thermal cycler in the following order: 27 °C for 10 min, 38 °C for 15 min, 44 °C for 40 min, 72 °C for 15 min. All the primers which were used to detect viral genes (E2, E6 and E7) are listed in a table in the (supplementary materials). To detect viral genes E2, E6 and E7, Quantitative SYBR green TaqMan Universal PCR Master Mix® (QIAGEN, Germany), one step RT-PCR® kits (QIAGEN, Hilden, Germany) and QuantiNova Reverse Transcription® Kit were used, respectively.

For viral genes we used serial dilutions of E2, E6 and E7 genes cloned in PUC57 vector (GenScript, Jiangsu, China). Serial dilution was containing equivalent amounts of these genes from 72 to 865 million copies per reaction, served as a standard control.

#### mRNA level detection of cellular genes

cDNA was synthesized using the PrimeScript First Strand cDNA synthesis kit (TaKaRa Bio, Kusatsu, Japan). Quantitative RT-PCR analyses were performed using the Power SYBR Green PCR Master Mix (TaKaRa Bio, Kusatsu, Japan). The relative expression level of each mRNA was normalized using *GAPDH*. The primers are listed in supplementary materials.

### Enzyme linked immunosorbent assay (ELISA)

For tissue homogenization, all fresh tissue samples were weighed and the tissue lysate was prepared according to the manufactures protocol (Invitrogen, CA, USA). Approximately 50 μg of each tissue was excised and washed with ice-cold PBS.

The level of p53, RB, IL-1, IL-6, IL-11, NF-kB, NF-κ PTPN13, E-cadherin, N-cadherin and TWIST was assessed using Abcam’s p53 Simple Step ELISA® Kit (Abcam, Cambridge, MA, USA), Human Retinoblastoma ELISA® kit (Sigma-Aldrich, Saint Louis, USA), Human Retinoblastoma ELISA® kit (Sigma-Aldrich, Saint Louis, USA), Human IL-6 ELISA® Kit, Human IL-1 beta ELISA® Kit, Human IL-11 ELISA® Kit, NF-kB p65 Transcription Factor Assay® Kit (Abcam, Cambridge, MA, USA), Human Tyrosine-Protein Phosphatase Non-Receptor Type 13 (PTPN13) ELISA Kit (MyBiosource, USA), Human E-Cadherin, N-Cadherin ELISA Kit (Abcam, Cambridge, MA, USA).and TWIST ELISA Kit (Aviva Systems Biology, CA, USA).

### Quantification of RONS

The RONS level was assessed by OxiSelect™ Intracellular ROS/RNS Assay kit (Cell Biolabs, Inc., San Diego, CA). For this purpose, cell lysate was used and preparation of this based on Kit instructions.

### Statistical methods

Continuous variables are presented as mean ± standard deviation and categorical variables are presented as N (%). Normality test was checked using Kolmogorov–Smirnov test for the continuous variables. For comparing the central tendency (e.g. mean for normal and median for non-normal variables) between two groups, two-independent samples t-test or Mann-Whitney non-parametric test and between more than two groups, one-way ANOVA or kruskal-wallis test were used. Chi-square/ or Fisher exact test was performed for assessing the associations of the categorical variables. The unit of all expression RT-PCR is (2^-DCt)*1000. Internal normalization was performed using an internal housekeeping or reference gene (GAPDH) and external normalization was applied by standardized approach. In addition, correlation analysis was done by Spearman’s correlation coefficient between viral and cellular factors. All of statistical analyses were analysed using GraphPad Prism 6 and STATA software versions 11.2. False discovery rate was corrected by Benjamini-Hochberg approach for multiple comparisons. A two-sided *P*-value of less than 0.05 was considered as statistical significance.

## Results

In this case-control study, we examined 102 lung cancer cases and 48 controls, with the mean ± SD age; 56.36 ± 12.49 and 57.0 ± 12.24, respectively. Seventy-four (72.5%) of the cases and 31 (64.5%) of the controls were male, respectively. The cases and control groups were matched based on age (*p* = 0.77). There were three types of lung tumour tissues; squamous-cell carcinoma (51.9%), adenocarcinoma (32.3%) and SCLC (15.7%). The highest and lowest stages of cancer in this study were IIIB (30.4%) and IA and IIB (1.9%) respectively. HPV DNA was detected in 52.9% of the lung cancer specimens and in 25% of control samples. There was a significant association between the presence of HPV and lung tumour (OR = 3.37, 95% C.I = 1.58–7.22, *P* = 0.001). Genotype 16 was the most frequently isolated genotype in both cases (38.8%) and controls (50%). No significant association was observed between all genotypes and the occurrence of lung tumour (*p* = 0.651) (supplementary materials). HPV DNA was detected in 55.6% (30 of 53) of squamous-cell carcinoma samples, 54.5% (18 of 33) of adenocarcinoma samples and 37.5% (6 of 16) of SCLC samples. The association between HPV infection and histopathological types of tumour was not statistically significant (*p* = 0.434). There were no significant differences in the frequency distributions of lung tumour stages between HPV+ and HPV- groups (*p* = 0.163). More information is presented in supplementary materials.

**Fig. 1:**
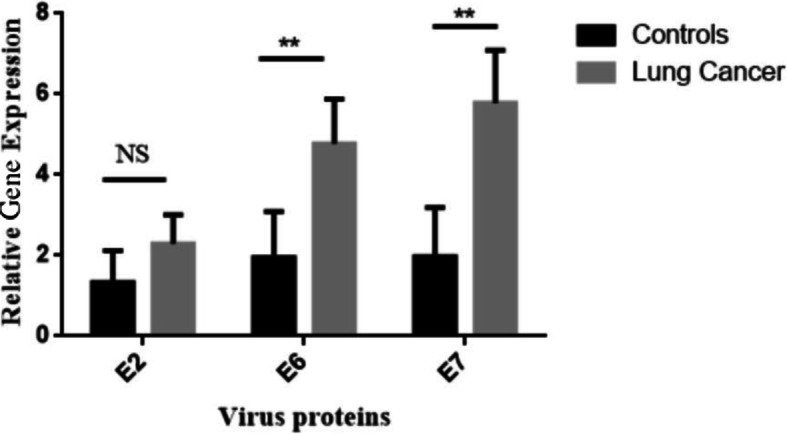
Comparison of *E2*, *E6*, and *E7* gene expression in lung cancer versus control. NS: not significant at level of 0.05. (** *P*<0.01)

In the HPV+ lung carcinoma patients, the virus was present in its integrated form in 27.8% of cases. The incidences of episomal and mixed forms of HPV genome were 5.5 and 66.7% respectively. In the control HPV+ group, the incidence of HPV genome status was 25, 0 and 75% integrated, episomal and mixed forms of HPV respectively (Table 1). The gene expression level of viral genes in both types and stages of lung tumour are shown in Table 2. The highest level of viral gene expression was that of *E7* which was most highly observed in stage IV samples (mean ± SD:13.56 ± 5.13). The lowest level of viral gene expression examined was *E6* in stage IB samples (mean ± SD: 3.0 ± 1.75). The gene expression level of viral factors *E2* and *E6* were highest in stage IIB and stage IV respectively. Stratification of the samples based on the tumour type reveals the expression level of *E7* in adenocarcinoma samples (mean ± SD: 11.94 ± 4.93) and *E2* in SCLC (mean ± SD: 3.67 ± 1.15) were the highest and lowest respectively (Table 2***)***. The expression level of viral genes in control samples and tumour samples are illustrated in Fig. 1.

**Table 1:** Physical status of HPV genome in cases and controls

	Cases (%)		Controls (%)	Total number (%)	***P***-value
**Integrated**	15/54 (27.8) Tumour Stages: IA (*N* = 0) IB (*N* = 2) IIA (*N =* 0) IIB (*N* = 3) IIIA (*N* = 1) IIIB (*N =* 3) IV (*N* = 6)	Tumour Types: Adenocarcinoma (*N* = 4) Squamous-cell carcinoma (*N =* 6) Small-cell lung carcinoma (*N* = 5)	3/12 (25)	18/66 (27.3)	0.845
**Episomal**	3/54 (5.5) Tumour Stages: IA (*N =* 0) IB (*N =* 1) IIA (*N =* 0) IIB (*N =* 2) IIIA (*N =* 0) IIIB (*N =* 0) IV (*N =* 0)	Tumour Types: Adenocarcinoma (*N =* 0) Squamous-cell carcinoma (*N =* 2) Small-cell lung carcinoma (*N =* 1)	0	3/66 (4.5)	NA
**Mixed**	36/54 (66.7) Tumour Stages: IA (*N =* 1) IB (*N =* 0) IIA (*N =* 4) IIB (*N =* 3) IIIA (*N =* 7) IIIB (*N =* 6) IV (*N* = 15)	Tumour Types: Adenocarcinoma (*N =* 5) Squamous-cell carcinoma (*N* = 22) Small-cell lung carcinoma (*N* = 9)	9/12 (75)	45/66 (68.2)	0.827

*NA* Not available

**Table 2:** Comparison of HPV gene expression between stages, types of lung cancer, and controls

Cancer characteristic		E2	E6	E7
**Controls (*n*** **= 48)**	–	5.82 ± 2.48 (1)	8.36 ± 3.14 (1)	8.64 ± 4.30 (1)
**Stages of Cancer (*n*** **= 102)**	IA	4.0 ± 1.27 (0.68)	9.0 ± 1.2 (1.07)	8.0 ± 1.89 (0.92)
	IB	5 ± 1.0 (0.86)	3.0 ± 1.75 (0.36)	5.0 ± 0.57 (0.58)
	IIA	8 ± 5.29 (1.37)	8 ± 2.45 (0.95)	9.75 ± 4.50 (1.13)
	IIB	6.83 ± 2.32 (1.17)	9.1 ± 4.77 (1.09)	10.8 ± 6.59 (1.25)
	IIIA	4.3 ± 3.02 (0.74)	8.54 ± 3.75 (1.02)	11.23 ± 4.95 (1.30)
	IIIB	6.58 ± 4.08 (1.13)	8.13 ± 5.23 (0.97)	10.13 ± 5.17 (1.17)
	IV	6.0 ± 3.83 (1.03)	12.33 ± 4.66 (1.47)	13.56 ± 5.13 (1.57)
	***P*-value**	0.585	0.301	0.643
**Types of Cancer (*n*** **= 102)**	Adenocarcinoma	5.83 ± 3.69 (1)	9.67 ± 4.99 (1.17)	11.94 ± 4.93 (1.38)
	Squamous-cell carcinoma	6.39 ± 3.69 (1.1)	8.63 ± 4.70 (1.03)	10.17 ± 5.57 (1.18)
	Small-cell lung carcinoma	3.67 ± 1.15 (0.63)	9.0 ± 3.28 (1.07)	11.67 ± 5.09 (1.35)
	***P*-value**	0.466	0.762	0.504

Geometric Mean ± Standard Deviation (fold change), control group was as a reference group

**Fig. 2:**
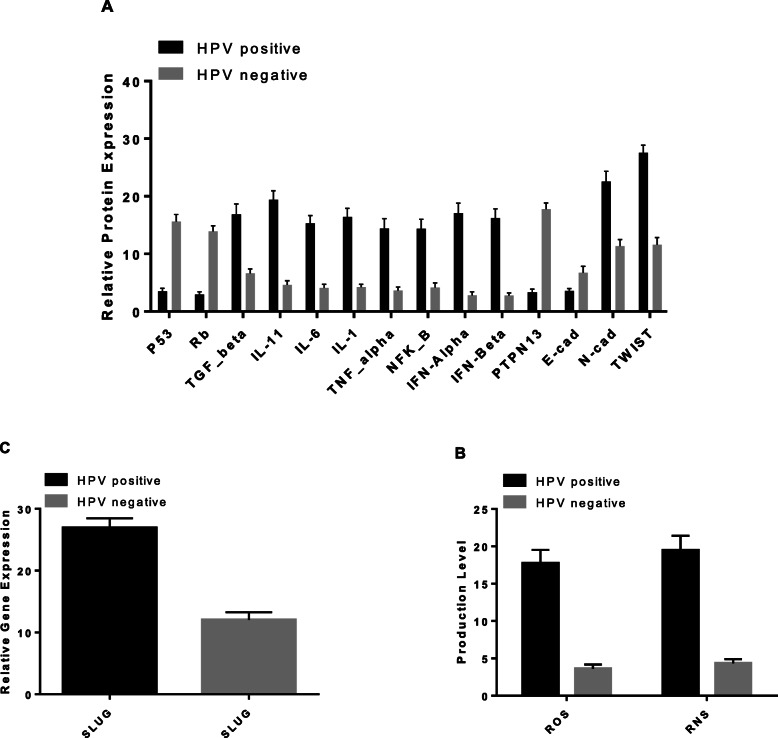
Comparison of the (**a**) cell factors expression, (**b**) ROS and RNS agents and (**c**) SLUG factors in HPV positive versus HPV negative subjects. All the statistical comparisons were significant at level of 0.001 by independent T-test

In Table 3, the level of cellular factors such as tumour-suppressors (Rb and p53), inflammatory factors (ILs, IFNs, TGF-β, TNF-α, and NF-κB), EMT factors (PTPN13, SLUG, E-cad, N-cad and TWIST) and RONS are presented. The protein levels of Rb and p53 were significantly downregulated in HPV+ cases and HPV+ controls compared with HPV- cases and controls (*p* < 0.001). The level of inflammatory factors, were considerably higher in HPV+ cases and controls compared to the HPV- cases and controls groups. The levels of EMT involved factors found to be significantly higher in HPV infected group compare to HPV non-infected group (*p* < 0.001 for all). Among the EMT involved genes, PTPN13 and E-cad were significantly downregulated in HPV+ cases and controls compared with HPV- cases and controls (*p <* 0.001). SLUG, N-cad and TWIST were significantly upregulated in HPV+ cases and controls compared with HPV- cases and controls (*p <* 0.05). The highest expression levels were related with SLUG, N-cad and TWIST in HPV+ compared with HPV- groups (fold change > 15; *p <* 0.001 for all). More details are presented in Fig. 2***.*** Significant negative correlations were observed between the expression level of viral genes and the protein expression levels of regulatory host proteins, Rb and p53. Among the inflammatory factors examined, the correlations between *E2* expression level with IL-1 and TNF-α were statistically significant, and the correlations between IL-6 with *E6* and *E7* were statistically significant (*p <* 0.01). The correlation between *E2* expression level and PTPN13 was positive but with SLUG, E-cad, N-cad and TWIST was negative. The expression level of *E6* significantly correlated with the protein level of PTPN13. The expression level of E7 has the negative correlation with E-cad and N-cad (*p <* 0.05). Conversely, there were positive correlations between *E6* gene expression and IL-1, IL-6, IFN-α and IFN-β protein levels and RONS production (*p <* 0.05) (Table 3).

**Table 3:** Comparison of cellular factors levels between the studied groups

Cellular factors	Patient with HPV + (***N*** = 54), Group 1	Patient with HPV - (***N*** = 48), Group 2	Control with HPV + (***N*** = 12), Group 3	Control with HPV - (***N*** = 36), Group 4	F.change, P^*^	F.change, P^**+**^	F.change, P^**$**^
**P53**	3.7 ± 2.01	16.02 ± 6.34	2.75 ± 1.54	15.11 ± 4.48	0.24, **< 0.001**	1.06, 0.677	0.18, **< 0.001**
**Rb**	2.94 ± 1.85	12.5 ± 3.68	3.0 ± 2.29	15.78 ± 4.89	0.18, **< 0.001**	0.79, **< 0.001**	0.19, **< 0.001**
**TGF_β**	18.57 ± 6.76	5.81 ± 3.27	9.33 ± 3.60	7.72 ± 3.29	2.4, 0.637	0.75, 0.189	1.2, **< 0.001**
**IL-11**	19.09 ± 6.08	5.25 ± 3.85	20.75 ± 7.34	3.78 ± 2.19	5.05, **< 0.001**	1.38, 0.382	5.49, **< 0.001**
**IL-6**	15.19 ± 5.30	3.88 ± 3.56	16.0 ± 6.03	4.33 ± 1.93	3.51, **< 0.001**	0.89, 0.931	3.69, **< 0.001**
**IL-1**	16.19 ± 5.88	4.04 ± 2.54	17.42 ± 7.26	4.44 ± 2.13	3.64, **< 0.001**	0.9, 0.957	3.92, **< 0.001**
**TNF_α**	12.83 ± 5.97	3.54 ± 3.33	21.5 ± 6.97	3.81 ± 1.43	3.36, **< 0.001**	0.93, 0.988	5.64, **< 0.001**
**NFK_B**	13.02 ± 5.89	4.15 ± 4.42	20.58 ± 6.54	4.17 ± 2.07	3.12, **< 0.001**	0.99, 0.99	4.93, **< 0.001**
**IFN-Alpha**	16.94 ± 7.37	2.92 ± 3.58	17.75 ± 5.31	2.67 ± 1.19	6.34, **< 0.001**	1.09, 0.993	6.64, **< 0.001**
**IFN-Beta**	16.09 ± 6.53	2.73 ± 2.37	16.83 ± 6.01	2.86 ± 1.39	5.62, **< 0.001**	0.95, 0.998	5.88, **< 0.001**
**PTPN13**	2.91 ± 1.53	19.33 ± 4.84	5.25 ± 3.52	15.66 ± 3.89	0.18, **< 0.001**	1.23, **< 0.001**	0.33, **< 0.001**
**E-cad**	3.61 ± 1.82	4.88 ± 3.55	3.42 ± 0.99	9.17 ± 6.05	0.39, **< 0.001**	0.53, **< 0.001**	0.37, **< 0.001**
**N-cad**	23.48 ± 7.09	9.83 ± 4.27	18.33 ± 7.49	13.36 ± 5.89	1.75, **0.041**	0.73, **0.025**	1.37, **0.001**
**TWIST**	28.43 ± 5.0	12.54 ± 5.50	23.58 ± 5.76	10.31 ± 6.23	2.75, **< 0.001**	1.22, 0.171	2.28, **0.001**
**ROS**	17.37 ± 7.57	3.65 ± 2.93	19.25 ± 6.81	3.58 ± 1.93	4.85, **< 0.001**	1.02, 0.99	5.38, **< 0.001**
**RNS**	19.19 ± 8.43	4.48 ± 3.07	20.58 ± 6.06	4.14 ± 1.98	4.63, **< 0.001**	1.08, 0.987	4.97, **< 0.001**
**SLUG**	28.33 ± 5.59	14.08 ± 5.47	20.42 ± 6.11	9.28 ± 4.94	3.05, **< 0.001**	1.52, **< 0.001**	2.2, **< 0.001**

F. change: Fold Change, Geometric Mean ± Standard Deviation, * comparison between group 1 versus group 4, + comparison between group 2 versus group 4, $ comparison between group 3 versus group 4. Control with HPV negative considered as the reference group. P is adjusted *P*-value based on the marginally adjusted 푝 values by the Benjamini-Hochberg-FDR correction at α = 0.05, Bold *P*-values indicated as statistically significant at 0.05 level

## Discussion

Lung cancer is the primary cause of cancer death globally [33]. As such, there is a major unmet clinical need for the development and discovery of prognostic biomarkers for the diagnosis of lung cancer. This need is underlined by the increased mortality rates which are currently being observed in lung cancer worldwide [1, 15]. A plethora of carcinogens are responsible for the initiation and development of various cancers. Of these, viral infections are implicated in approximately 18–20% of cancers [6, 11, 34]. While the prevalence of HPV in lung carcinoma has shown in numerous studies, to date, the role of hr-HPV in the promotion of EMT has not yet been clearly identified. Here, we report for the first time the association between HPV gene expression, inflammatory agents and cellular genes involved in EMT in lung cancer tissue.

In the current study, 52.9% of lung tumour samples were positive for HPV. Moreover, we demonstrate that increased expression of HPV genes is associated with decreased expression of regulatory cellular genes, RB and p53, and as a result increased risk of lung cancer. In an investigation Nadji et al. (2007, Iran) studied 141 lung carcinoma samples and 92 non-cancersamples as controls. Results demonstrated that 25.6% of cases and 9.0% of controls were positive for HPV infection respectively. The reported odds ratio for HPV infection was 3.48 (95% CI 1.522–7.958; *P* = 0.002) [3]. A similar study by Argyria et al. (2017, Greece) investigated 67 lung tissue samples from a Greek cohort, 12 SCLC and 55 NSCLC, and detected HPV in 3.0% of the samples; with no association between HPV infection and lung cancer. Other studies conducted in this region showed the prevalence of HPV DNA rate was 0–61% [10]. Robinson et al. (2016, USA) examined 70 NSCLC samples for detection of viral DNA. They detected 69% of HPV DNA in their samples [35]. A meta-analysis study which performed by Syrjanen and his research team (2012) showed the prevalence of HPV in Europe (16.9%) in Australia (18.5%), in North America (12.5%) and in China and Taiwan (37.7%). This study shows the role of geographical distribution in HPV prevalence [36]. Another point in the current study, was the highest SCC tumour type (51.9%) that is confirmed by a meta-analysis study (Almasi et al. 2016, Iran) [37]. The most common HPV genotypes detected in cancer patients are HPV 16 and 18 [6, 38–40]. Previous investigations have been confirmed this issue and also, a higher prevalence of HPV-16 and 18 in Asian populations compared with European populations (lung samples). It should be noted that HPV-16 is the most prevalent genotype across all geographical areas [38]. In another interesting study, the presence of HPV DNA was examined in the exhaled breath condensate (EBC) of lung cancer patients by Carpagnano et al. (2011, Italy). Their results showed the presence of HPV infection in 16.4% of samples. The authors state that analysis of EBC for HPV infection represents a valid tool for the diagnosis of airway colonisation [41].

Previous investigations have noted the physical status of HPV DNA as an important marker for tumour progression in other cancers, such as breast cancer [32]. In this study, the highest integrated form was seen in stages III and IV in SCC samples (Table 1). Khodabandehlou et al. previously reported on the physical status of HPV genome in breast cancer samples, with 86% integrated and 14% mixed forms respectively. The largest number of integrated forms was in stage III and IV [6]. Detection of HPV in its integrated form has also been reported in several other cancers [30, 42, 43]. The integration of HPV genome leads to changes in the expression of viral oncogenes (E6 and E7), dysregulating of critical cell cycle checkpoints, increased genetic instability in the host and finally tumour development [44].

We examined the potential role of HPV in lung cancer pathogenesis in two ways: i) the impact of HPV on the expression of genes involved in EMT, ii) the impact of HPV in the development of chronic inflammation and microenvironment alteration. EMT promotes cancer development through enhancing cellular migration and invasion [25, 45]. Oncoviruses are said to promote EMT in particular cancer cells, enabling the spread of metastatic cells from one location to another [21]. Hr-HPV interacts with EMT factors to promote tumour development. Our results demonstrate that the levels of genes which promote EMT were substantially higher in HPV positive groups compare to HPV negative groups (*p* < 0.001 for all) (Table 2). This situation could indicate that the viral genes products/proteins may be involved in stimulating of transcription of these genes. We have hypothesized that hr-HPV promotes lung cancer development indirectly through a variety of different mechanisms. For example, HPV induce the production of ROS that leads to cell survival and resistance to programmed cell death [46]. Previous studies have shown that in lung cancers with impaired E-cadherin expression, the frequency of lymph node metastases was significantly higher than tumours with high expression of the E-cadherin [28, 47, 48]. In our study, expression of E-cadherin in HPV+ samples were lower than HPV-negative samples (Fig. 2), with viral *E7* detection having a negative correlation with E-cadherin levels (Table 2). Unlike E-cadherin, protein levels of N-cadherin in HPV+ samples were higher than HPV-negative samples; which has previously been shown to be associated with tumour development [49, 50]. On the other hand, TGF-β lead to an increase of N-cadherin and the expression of TGF-β in HPV+ samples was higher than HPV-negative samples (Fig. 2***,*** Table 3) [50]. Another important cellular factor is SLUG. This protein is overexpressed in numerous cancers [51]. High expression levels of SLUG has also been shown to be associated with reduced E-cadherin expression, high histologic grade, lymph node metastasis, postoperative relapse and shorter survival in patients with cancer [51–53]. SLUG also has a role in inflammation-dependent tumour development [54]. Our results demonstrate that the gene expression level of this factor was higher in HPV+ samples than HPV-negative samples (Fig. 2***,*** Table 3). Furthermore, expression levels of SLUG have been shown to correlate with lung tumour development and drug resistance [55]. Our results show the over expression of SLUG in HPV+ samples and direct correlation with *E6* and *E7* (Tables 2 and 4).

**Table 4:** Spearman’s correlation coefficient between viral factors and cell factors

	E2	E6	E7
**P53**	−0.19	−0.44^**^	−0.34^**^
**Rb**	−0.01	−0.36^**^	−0.46^**^
**TGF-β**	−0.14	−0.11	−0.04
**IL-11**	−0.27	0.22	0.19
**IL-6**	0.25	0.40^**^	0.35^**^
**IL-1**	0.47^**^	0.31^*^	0.30^*^
**TNF_α**	−0.36^*^	0.03	0.05
**NFκB**	−0.27	0.09	0.11
**ROS**	0.09	0.36^**^	0.39^**^
**RNS**	0.18	0.33^**^	0.34^**^
**IFN-Alpha**	0.02	0.27^*^	0.35^**^
**IFN-Beta**	0.14	0.35^**^	0.27^*^
**PTPN13**	0.09	−0.10^*^	−0.21
**SLUG**	−0.02	0.08	0.18
**E-cad**	−0.13	−0.24	− 0.21^*^
**N-cad**	−0.19	− 0.12	−0.08^*^
**TWIST**	−0.13	−0.02	0.05

* *p* < 0.05; ** *p* < 0.01; *** *p* < 0.001

The tumour microenvironment is a key factor in tumour development and several epidemiologic and clinical researches have proposed a strong association between inflammation related to chronic infection and lung cancer [20, 56–58]. This inflammation affects different aspects of tumor development such as angiogenesis, survival of malignant cells and even tumor response to therapy [59, 60].

Our results demonstrate that the expression of numerous inflammatory factors was higher in HPV+ samples than HPV-negative samples (Table 3). Previously, Stone et al. (2014, Brazil) have shown HPV dependant changes in the tumour microenvironment. Their results showed differences in local inflammation between HPV+ and HPV-negative tumour tissues [56]. Liu et al. (2015, China) have also studied the association between HPV and chronic inflammation, demonstrating that chronic inflammation was higher in oropharyngeal tumour tissue compared to normal tissues (*P* < 0.001). They propose that HPV infection could be considered as a biomarker/risk in some cancers in individuals with chronic inflammation [61]. Previous investigations have shown microenvironmental alterations, caused by microorganisms, such as cytokine secretion promote epithelial proliferation. This issue was demonstrated in HPV infection and its persistence, which increases the risk of HPV transmission and oropharyngeal carcinogenesis [62–64].

In the current study the highest expression level of viral genes was in stage IV and the lowest level was in Stage IA and IB. In the other words, viral genes can be related to chronic inflammation and EMT (Table 2). This issue indicates the important role of these gene products in tumour development and metastasis. Although HPV is an oncovirus, the presence of the virus alone is insufficient for tumorigenesis. In order to promote cancer development, it is necessary to have a proinflammatory tumour microenvironment which occurs due to exposure to environmental factors or altered immunological mechanisms [20]. Also, should be noted that the possibility to get HPV after premalignant lesions appear and how this concomitant infection may promote cancer progression but not lung cancer origin.

A key risk factor associated with HPV infection is smoking status. Previous studies have demonstrated the relationship between smoking and HPV infection in some cancers such as lung and cervical [1, 65]. In an investigation, relationship between HPV infection and cigarette smoking was studied (by Xi et al.). They demonstrated that HPV DNA load (type 16, 18) was associated with status of smoking, and current smokers had a higher HPV DNA load compared to former smokers [65].

Limitation of the current study were including:

i) Limitations on the number of cases considered for the study and the lack of statistical representation for some tumor stages; ii) protein and RNA samples are pooled representation of the different cell types from the original tumor/control tissue; iii) the absence of medical information regarding a HPV infection before cancer diagnosis for the patients analyzed in this research.

## Conclusion

In summary, the presence of HPV was detected in 52.9% of lung cancer samples among which most were at stage III and IV (73.5%). Infection of HPV directly promotes local inflammation which in turn promotes tumorigenesis and cancer development. We demonstrate that HPV is associated with lung cancer development, although the role of hr-HPV in lung cancers requires further study. To the best of our knowledge, this is the first study reporting the role of HPV genes expression in EMT and the association between this virus and chronic inflammation in lung cancer patients.

## Supplementary information

**Additional file 1.**

## Data Availability

The datasets used and/or analyzed during the current study could become available through the corresponding author on reasonable request.

## Abbreviations

HPV: Human papillomavirus
RB: Retinoblastoma
SCC: Squamous cell carcinoma
SCLC: Small cell lung cancer
NSCLC: Non-small cell lung cancer
IPF: Idiopathic Pulmonary Fibrosis
COPD: Chronic Obstructive Pulmonary Disease
LCR: Long control region
TNF-α: Tumour necrosis factor α
RONS: Reactive oxygen-nitrogen species
TGF-β: Transforming growth factors like beta
ECM: Extracellular matrix
NF-κB: Nuclear factor kappa beta
EMT: Epithelial-mesenchymal transition.
