# Supplementary material for: The association between HPV gene expression, inflammatory agents and cellular genes involved in EMT in lung cancer tissue
Source: BMC Cancer. 2020 Sep 24;20:916. doi: 10.1186/s12885-020-07428-6 (PMC7517685; doi:10.1186/s12885-020-07428-6)
Supplement: Supplementary file 1 — Additional file 1. [file 12885_2020_7428_MOESM1_ESM.doc]

Table 1. Primers and probes for gene amplification.

|  | Primers and Probes Sequences | Ref |
| --- | --- | --- |
| E2 | 5'-CTACGAATTCATGGAGACTCTTTGCCAACG-3′  5'-GATAGAATTCTCATATAGACATAAATCCAG-3′ | (1) |
| E6 | 5'-GCAATGTTTCAGGACCCACA-3'  5'-ACAGCATATGGATTCCCATCTC-3' | (2) |
| E7 | 5'-AAGTGTGACTCTACGCTTCGGTT-3'  5'-GCCCATTAACAGGTCTTCCAAA-3'  Probe: FAM-TGCGTACAAAGCACACACGTAGACATTCGTA-BHQ | (3) |
| SLUG | 5'-GCCTCCAAAAAGCCAAACTACA-3'  5'-GAGGATCTCTGGTTGTGGTATGACA-3' | (4) |
| GAPDH | 5'- ACACAACTGTGTTCACTAGC -3'  5'-CAACTTCATCCACGTTCACC -3' | (5) |

Table 2. Comparison of participant’s characteristics between lung cancer, control, HPV positive and negative groups.

| **Characteristics** | | **Lung Cancer (n=102)** | **Control (n=48)** | **P-value^*^** | **HPV +** | **HPV -** | **P-value^$^** |
| --- | --- | --- | --- | --- | --- | --- | --- |
| **Age (Year)** | | 56.36 ± 12.49 | 57.0 ± 12.24 | 0.770 | 56.51 ± 12.54 | 56.64 ± 12.26 | 0.952 |
| **Sex** | Male | 74 (72.5%) | 31 (64.5%) | 0.321 | 49 (74.2%) | 56 (66.6%) | 0.315 |
|  | Female | 28 (27.5%) | 17 (35.5%) |  | - | - |  |
| **HPV** | Presence | 54 (52.9%) | 12 (25%) | **0.001** | - | - | NA |
|  | Absence | 48 (47.1%) | 36 (75% |  | - | - |  |
| **Smoking** | Yes | 72 (70.5%) | 11 (22.9%) | **0.001** | 35 (53.0%) | 25 (29.7%) | 0.126 |
|  | No | 30 (29.5%) | 37 (77.1%) |  | - | - | - |
| **Genotype** | 6 | 4 (7.4%) | 1 (8.3%) | 0.651 | - | - | NA |
|  | 11 | 4 (7.4%) | 2 (16.6%) |  | - | - |  |
|  | 16 | 21 (38.8%) | 6 (50%) |  | - | - |  |
|  | 18 | 20 (37.03%) | 2 (16.6%) |  | - | - |  |
|  | 33 | 5 (9.3%) | 1 (8.3%) |  | - | - |  |
| **Stage of Cancer** | IA | 2 (1.9%) | - | NA | 1 (1.85) | 1 (2.08%) | 0.163 |
|  | IB | 2 (1.9%) | - |  | 1 (1.85%) | 1 (2.08%) |  |
|  | IIA | 8 (7.8%) | - |  | 4 (7.40%) | 4 (8.33%) |  |
|  | IIB | 15 (14.7%) | - |  | 10 (18.51%) | 5 (10.41%) |  |
|  | IIIA | 18 (17.6%) | - |  | 13 (24.07%) | 5 (10.41%) |  |
|  | IIIB | 31 (30.4%) | - |  | 16 (29.62%) | 15 (31.25%) |  |
|  | IV | 26 (25.5%) | - |  | 9 (16.66%) | 17 (35.41%) |  |
| **Type of Cancer** | Adenocarcinoma | 33 (32.3%) | - | NA | 18 (33.33%) | 15 (31.25%) | 0.434 |
|  | Squamous-cell carcinoma | 53 (51.9%) | - |  | 30 (55.55%) | 23 (47.91%) |  |
|  | Small-cell lung carcinoma | 16 (15.7%) | - |  | 6 (11.12%) | 10 (20.83%) |  |

NA: Not available.

**References**

1. Webster K, Parish J, Pandya M, Stern PL, Clarke AR, Gaston K. The human papillomavirus (HPV) 16 E2 protein induces apoptosis in the absence of other HPV proteins and via a p53-dependent pathway. Journal of Biological Chemistry. 2000;275(1):87-94.

2. Jang M, Rhee J, Jang D-H, Kim SS. Gene expression profiles are altered in human papillomavirus-16 E6 D25E-expressing cell lines. Virology journal. 2011;8(1):453.

3. Wang‐Johanning F, Lu DW, Wang Y, Johnson MR, Johanning GL. Quantitation of human papillomavirus 16 E6 and E7 DNA and RNA in residual material from ThinPrep Papanicolaou tests using real‐time polymerase chain reaction analysis. Cancer. 2002;94(8):2199-210.

4. Nakamura R, Ishii H, Endo K, Hotta A, Fujii E, Miyazawa K, et al. Reciprocal expression of Slug and Snail in human oral cancer cells. PloS one. 2018;13(7):e0199442.

5. Khan N, Castillo A, Koriyama C, Kijima Y, Umekita Y, Ohi Y, et al. Human papillomavirus detected in female breast carcinomas in Japan. British journal of cancer. 2008;99(3):408.
